# Smartphone-based ecological momentary assessment reveals mental health benefits of birdlife

**DOI:** 10.1038/s41598-022-20207-6

**Published:** 2022-10-27

**Authors:** Ryan Hammoud, Stefania Tognin, Lucie Burgess, Nicol Bergou, Michael Smythe, Johanna Gibbons, Neil Davidson, Alia Afifi, Ioannis Bakolis, Andrea Mechelli

**Affiliations:** 1 Department of Psychosis Studies, Institute of Psychiatry, Psychology and Neuroscience, King’s College London, De Crespigny Park, London, SE5 8AF UK; 2 Nomad Projects, Sunbury Workshops, 24, Swanfield St, London, E2 7LF UK; 3 J&L Gibbons, 19 Swan Yard, London, N1 1SD UK; 4 Health Services and Population Research Department, Centre for Implementation Science, Institute of Psychiatry, Psychology and Neuroscience, King’s College London, London, UK; 5 Department of Biostatistics and Health Informatics, Institute of Psychiatry, Psychology and Neuroscience, King’s College London, London, UK

**Keywords:** Epidemiology, Health policy, Lifestyle modification, Psychology, Human behaviour

## Abstract

The mental health benefits of everyday encounters with birdlife for mental health are poorly understood. Previous studies have typically relied on retrospective questionnaires or artificial set-ups with little ecological validity. In the present study, we used the Urban Mind smartphone application to examine the impact of seeing or hearing birds on self-reported mental wellbeing in real-life contexts. A sample of 1292 participants completed a total of 26,856 ecological momentary assessments between April 2018 and October 2021. Everyday encounters with birdlife were associated with time-lasting improvements in mental wellbeing. These improvements were evident not only in healthy people but also in those with a diagnosis of depression, the most common mental illness across the world. These findings have potential implications for both environmental and wildlife protection and mental healthcare policies. Specific measures, aimed at preserving and increasing everyday encounters with birdlife in urban areas, should be implemented.

## Introduction

A growing body of empirical evidence is revealing the benefits of nature for mental health, including higher mental wellbeing and lower risk of mental illness^1^. The vast majority of the literature has focussed on the value of regular contact with green spaces including forests, parks, gardens and trees^2^. A smaller portion of the literature has investigated the value of blue spaces, including visible bodies of water such as lakes, rivers, canals, ponds and seas^2^. While these studies have led to growing appreciation of the mental health benefits of nature in general, we know little about the specific features within green and blue spaces which are driving these benefits. The present investigation focuses on a feature of the natural environment which has captivated humans over the centuries and yet has received very little scientific attention: birdlife.

In the United Kingdom, our captivation with birds is underlined by the fact that over 1.3 million people are members of the Royal Society for the Protection of Birds (more than the members of all political parties combined). Interest in birds is not a uniquely British phenomenon. In the United States, for example, over 70 million people are interested in birdwatching, making this one of the most popular nature-based recreational activities^3^. Birdwatching societies are not a phenomenon unique to the western world but are present worldwide, including countries with very different traditions and cultures. Despite the human fascination with birdlife, few studies have specifically examined the impact of encountering birds as part of everyday life on our mental health.

Using semi-structured interviews, Ratcliffe and colleagues reported that most people experience birdsong as restorative from psychological stress and attentional fatigue^4^. In a follow-up investigation using quantitative measures, the same authors reported that specific qualities of bird sounds such as perceived familiarity, complexity and pattern are predictive of perceived restorative potential^5^. Our encounters with birdlife, however, are multi-sensory experiences, typically involving both auditory and visual modalities in various degrees. It follows that both modalities need to be considered when assessing the possible benefits of birdlife for mental health. Consistent with this idea, a recent investigation showed that the perceived restorative potential of natural features such as wetland paths are enhanced by the presence of birdsong^6^.

The existing literature on the benefits of birdlife for mental health, however, is hindered by a number of methodological limitations. The majority of studies used surveys or questionnaires requiring participants to recollect past experiences^7^. We know that such method of investigation is susceptible to recall bias, especially in the case of people with mental illness^8^. The rest of the studies used an artificial set-up which involved the presentation of images or sounds of birds to people sitting in front of a computer screen. This is susceptible to generating data with little ecological validity, making it difficult to estimate the mental health benefits of seeing or hearing birds in real-life. A further limitation of the studies published so far is the limited sample size, with the vast majority of studies recruiting up to 30 participants.

To overcome the limitations of the existing literature, we assessed the association between seeing or hearing birds and self-reported mental wellbeing, a strong predictor of mental health in the general population^9^, using a smartphone-based application which we have developed^10^. The Urban Mind application (https://www.urbanmind.info/) uses Ecological Momentary Assessment (EMA), a methodology which involves sampling people’s experiences in real-time and in real-world contexts^11^. This methodology allowed us to explore the relationship between the experience of seeing or hearing birds and mental wellbeing while minimising the risk of recall bias. The Urban Mind application also collected detailed information on the participants, allowing us to explore how the impact of seeing or hearing birds on mental wellbeing depends on personal characteristics such as age, gender and having a diagnosis of mental illness. In particular we focussed on depression, which is the most common mental illness across the world and is predicted to become the first cause of global burden of disease by 2030^12^. Previous studies using surveys or questionnaires have reported a protective effect of green spaces on lifetime risk of depression^13,14^. However, no previous investigation has examined the mental health benefits of everyday encounters with birdlife in people with a diagnosis of depression. A better understanding of the effects of personal characteristics such as age, gender and having a common mental illness is critical for planning and designing urban and rural environments which support mental wellbeing in all citizens.

Using smartphone-based EMA, we carried out a citizen science study which aimed to answer the following questions:

1. Are encounters with birds as part of everyday life associated with higher mental wellbeing?
2. Is the beneficial impact of everyday encounters with birds on mental wellbeing time-lasting?
3. Does the beneficial impact of everyday encounters with birds on mental wellbeing differ between people with a diagnosis of depression and people without a mental health condition?

We hypothesised that everyday encounters with birds would be associated with higher mental wellbeing (hypothesis 1), and that this effect would still be evident after the encounter has taken place (hypothesis 2) and would be replicated in people with a diagnosis of depression and people without a mental health condition (hypothesis 3).

## Methods

The current study received institutional review board (IRB) approval from the Psychiatry, Nursing and Midwifery Research Ethics Subcommittee at King’s College London (LRS-17/18-6905). All research was performed in accordance with relevant guidelines and regulations.

### Design

Observational study using ecological momentary assessments in a smartphone application.

### Urban Mind app

**Figure 1:**
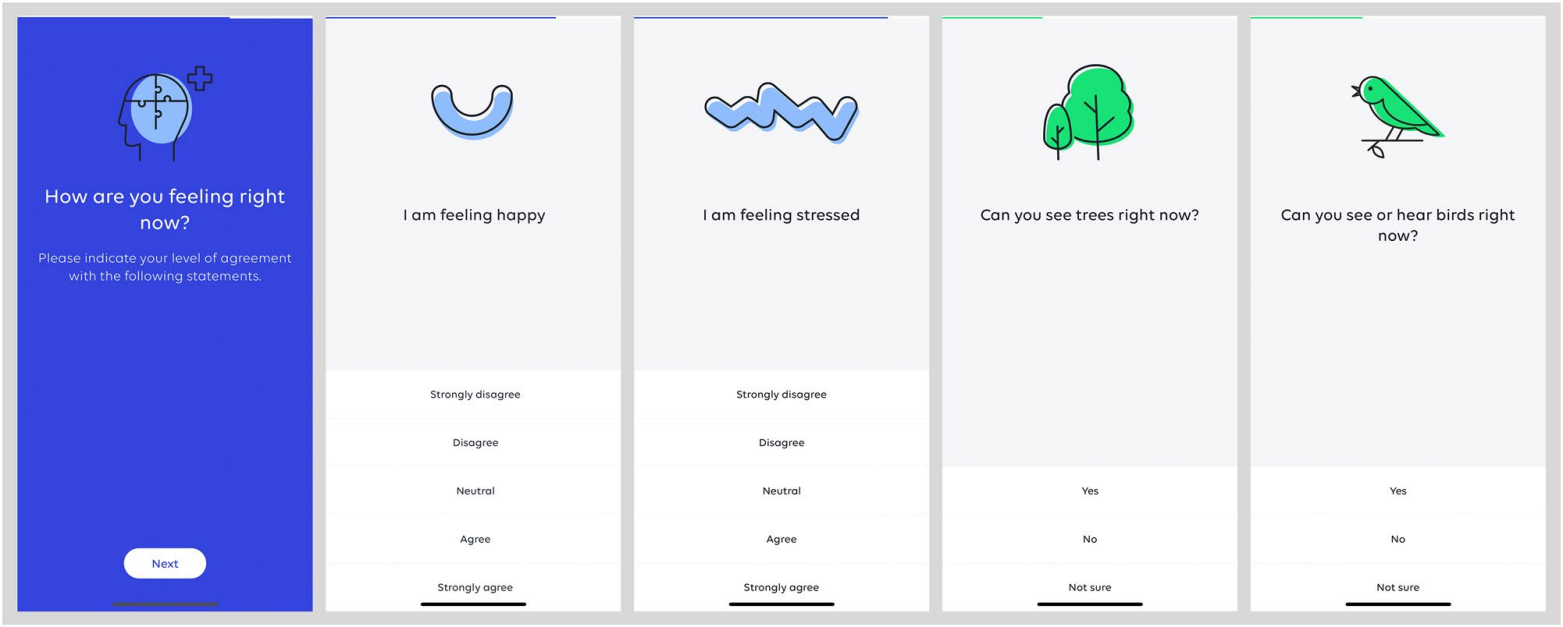
Screenshots of the Urban Mind app interface.

The present study was conducted using data collected from the Urban Mind smartphone application^10^ a smartphone-based EMA tool available for both Apple iPhone and Android devices. The app was developed as part of the Urban Mind research project—a collaboration between King’s College London, landscape architects J&L Gibbons and arts foundation Nomad Projects. Participants were recruited globally over a period of 42 months (April 2018–October 2021) using various social media platforms, the project-related website, and word of mouth. Participation in the study was self-selected and anonymous. Once an individual downloaded and installed the app, they were presented with information about the study, including the privacy policy about how their data would be used and were asked to provide informed consent. After consent was provided, participants were requested to complete a baseline assessment. This baseline assessment collected information regarding demographics (e.g. age, gender, ethnicity), socioeconomics (e.g. education, occupation), sleeping patterns (e.g. usual wake and sleep times) and self-reported mental health history (e.g. current and past mental health diagnoses). Following the baseline assessment, the application scheduled a total of 42 ecological momentary assessments during the following 14 days (3 assessments per day). As part of the baseline assessment, each participant was asked about their typical sleep pattern. The timeframe when they were awake was then divided into 3 equal windows, and the assessments were randomly scheduled within each window. Once an assessment was available, the app would prompt a participant to respond within 1 hour before the assessment was marked as incomplete. This allowed users to complete the assessment as they go about their daily lives, while minimising interruptions to any activities they were engaged in. Each EMA collected information about an individual’s perceived natural, built and social environment and their momentary mental wellbeing. The Urban Mind app is available in 8 languages including English, Cantonese, Mandarin, German, French, Spanish, Italian and Portuguese. Iconography is used heavily throughout the interface to improve engagement and ease of use (Fig. 1).

### Participants

A total of 1292 participants were included in our investigation. All participants completed at least 25% of the assessments (a minimum of 11 out of 42 assessments), 593 participants completed at least 50% of the assessments (21 out of 42 assessments), and 196 participants completed at least 75% of the assessments (32 out of the 42 assessments). A total of 2245 participants were excluded from the statistical analyses because they completed less than 25% of the ecological momentary assessments (fewer than 11 out of 42 assessments). Participants were based across the world, with the majority of them located in the United Kingdom (46%), the European Union (16%), the United States of America (8%), China (4%), and Australia (2%).

### Measures

#### Participant characteristics

Demographic characteristics were measured by asking participants the following questions: ‘How old are you?’, ‘What is your gender?’, ‘What is your ethnicity?’, ‘What is your highest level of education?’, and ‘What is your occupation?’. The presence of a diagnosis of depression was assessed by asking participants whether a doctor had ever diagnosed them with a mental health condition. When participants responded “Yes”, they were asked to indicate the specific diagnosis from a list of possible mental illnesses.

#### Encounters with birds

Within each EMA, encounters with birds as part of everyday life were assessed using a single item: “Can you see or hear birds right now?”. Participants could respond with either “No”, “Not sure” or “Yes”. The answers were recoded as a binary variable within the statistical analyses, with “No” and “Not sure” combined into a single category and “Yes” as the other category. This approach was followed due to the very small number of “Not sure” responses and was consistent with previous studies^10^.

#### Mental wellbeing

Current mental wellbeing was measured using 10 questions with 5-point Likert scale (Strongly Disagree, Disagree, Neutral, Agree, Strongly Agree). Five questions referred to positive mental wellbeing: I am feeling confident/ relaxed/ happy/ connected to other people/ energetic. Five questions referred to negative mental wellbeing: I am feeling anxious/ stressed/ down/ lonely/ tired. Questions referring to negative mental wellbeing were reverse-scored and added to the positive mental wellbeing scores, summing to a total mental wellbeing score between 10 (minimum) and 50 (maximum).

### Statistical analysis

All statistical analyses were performed with STATA/MP 16. Longitudinal associations between seeing / hearing birds and mental wellbeing were investigated using random intercept multilevel regression models, as appropriate for hierarchical longitudinal data. These longitudinal associations were expressed as mean difference (MD) and 95% confidence intervals (CI) of mental wellbeing. All models were adjusted for the following potential confounders: age, gender, ethnicity, level of education, occupational status, whether a participant could see trees, plants, and see or hear water. Due to small cell numbers, for the purpose of the statistical analysis, ethnic categories were collapsed into White, Asian (including Bangladeshi, Chinese, Indian, Pakistani, Japanese, Taiwanese, and Other Asian), and Other (Black, Caribbean, Latino or Hispanic, Indigenous, Arab, Other People of Colour and Mixed).

The main statistical analysis focused on individuals who had completed at least 50% of the assessments (*n* = 593), with sensitivity analyses on different priori-defined samples involving participants who had completed at least 25% of the assessments (*n* = 1,292) and those who completed at least 75% of the assessments (*n* = 196). The analyses were run as univariate models unadjusted for confounders and again as multivariate models adjusted for confounding variables. All models were rerun with the following modifications: (1) inclusion of the random slopes of the predictor variable and (2) within-subject centering of the predictor variables in line with best practice for multilevel models^15^. The results of these sensitivity analyses were then compared with the results of the main statistical analysis. Furthermore, in order to address missing data issues due to skipped assessments, all models were rerun using the STATA ice routine, an implementation of the Multiple Imputations with Chained Equations (MICE) procedure^16^. While this does not increase the tested sample sizes, this allowed us to impute the missing assessments from our samples. Our results using the MICE procedure were then compared with our results with the original analysis under the missing at random (MAR) assumption^17^, which assumes that the probability of missing assessments may differ between participants due to observed variables. Interaction effects between encounters with birds and diagnosis of depression on mental wellbeing was assessed by including interaction terms in the models. All results were considered significant if p < 0.05.

## Results

### Participant characteristics

Our total sample of 1292 participants included 912 (71%) females, 367 (28%) males, 11 (1%) other, and 2 preferred not to disclose, with an average age of 34.5 years (age range: 16–80). The sociodemographic characteristics of participants are presented in Table 1. The main statistical analysis focused on individuals who had completed at least 50% of the assessments (*n* = 593). This sample included 421 (71%) females, 168 (28%) males, 2 (0.3%) other and 2 who had preferred to not disclose their gender, with an average of 35.5 years (age range: 16–73). The sociodemographic characteristics of this sample and our two sensitivity samples are presented in Table 1.

**Table 1:** Sociodemographic characteristics of participants.

Assessment response rate	≥ 25% completion rate	≥ 50% completion rate	≥ 75% completion rate
	Number (%)	Number (%)	Number (%)
Number of participants	*n* = 1,292	*n* = 593	*n* = 196
Age in years	Mean: 34.5 SD: 13.3	Mean: 35.5 SD: 13.8	Mean: 36.7 SD: 15.0
**Gender**			
Female	912 (70.5%)	421 (71.3%)	135 (69.2%)
Male	367 (28.4%)	168 (28.4%)	60 (30.8%)
Other^a^	11 (0.85%)	2 (0.3%)	–
**Ethnicity**			
White	881 (68.4%)	413 (69.6%)	137 (69.9%)
Asian^b^	185 (14.4%)	81 (13.7%)	32 (16.3%)
Other^c^	221 (17.2%)	99 (16.7%)	27 (13.8%)
**Education**			
Less than high school	19 (1.5%)	10 (1.7%)	1 (0.5%)
High school	148 (11.5%)	66 (11.1%)	24 (12.2%)
Apprenticeship	88 (6.8%)	46 (7.8%)	15 (7.7%)
University	1037 (80.2%)	471 (79.4%)	156 (79.6%)
**Employment**			
Student	376 (29.1%)	171 (28.8%)	56 (28.6%)
Employed	691 (53.5%)	324 (54.6%)	103 (52.6%)
Self-employed	122 (9.4%)	47 (7.9%)	16 (8.2%)
Retired	46 (3.6%)	25 (4.2%)	14 (4.1%)
Unemployed	57 (4.4%)	26 (4.4%)	7 (3.5%)
**Diagnosis of mental health condition**			
No diagnosis	917 (71.0%)	418 (70.5%)	139 (70.9%)
Depression	246 (19.0%)	114 (19.2%)	37 (18.9%)
Other diagnoses	129 (10.0%)	61 (10.3%)	20 (10.2%)

Assessment response rate	≥ 25% completion rate	≥ 50% completion rate	≥ 75% completion rate
	Observations (%)	Observations (%)	Observations (%)
Number of momentary assessments	*n* = 26,856	*n* = 16,758	*n* = 6786
**Seeing/hearing birds**			
No/not sure	19,596 (73.0%)	12,232 (73.0%)	4833 (71.2%)
Yes	7257 (27.0%)	4524 (27.0%)	1953 (28.8%)
**Seeing trees**			
No/not sure	11,709 (43.6%)	7160 (42.7%)	2880 (42.4%)
Yes	15,147 (56.4%)	9598 (57.3%)	3906 (57.6%)
**Seeing plants**			
No/not sure	10,476 (39.0%)	6480 (38.7%)	2628 (38.7%)
Yes	16,381 (61.0%)	10,279 (61.3%)	4158 (61.3%)
**Seeing or hearing water**			
No/not sure	23,864 (89.0%)	14,888 (88.9%)	5903 (87.0%)
Yes	2987 (11.0%)	1867 (11.1%)	883 (13.0%)
Mental wellbeing	Mean: 34.7 SD: 7.3	Mean: 35.0 SD: 7.3	Mean: 35.6 SD: 7.2

Numbers and percentages may not add up due to missing values.

^a^Other: non-binary and other.

^b^Asian: including Bangladeshi, Chinese, Indian, Pakistani, Japanese, Taiwanese, and Other Asian.

^c^Other: including black, caribbean, latino or hispanic, indigenous, arab, other people of colour and mixed.

### Are encounters with birds as part of everyday life associated with higher mental wellbeing?

Multilevel regression analyses showed significant positive associations between seeing or hearing birds and mental wellbeing. The mean differences (MD) in the mental wellbeing scores were 1.72 (95% CI 1.53, 1.91) within our main sample, 1.53 (95% CI 1.37, 1.68) and 1.72 (95% CI 1.42, 2.02) at the sensitivity 25% and 75% response rates. The associations remained significant after adjusting for potential confounders (age, gender, ethnicity, education, occupation, whether a participant could see trees, plants, and see or hear water at the time of the assessment), after within-subject centering and including random slopes for the predictor variable, and after implementing the MICE procedure to address missing data (see Table 2, Supplementary Tables S1, S4, and S5).

**Table 2:** Associations between seeing or hearing birds and subjective mental wellbeing and interactions with depression.

	25% response rate (*n* = 1292)		50% response rate (*n* = 593)		75% response rate (*n* = 196)	
	Unadjusted	Adjusted	Unadjusted	Adjusted	Unadjusted	Adjusted
	MD (95% CI)	MD (95% CI)	MD (95% CI)	MD (95% CI)	MD (95% CI)	MD (95% CI)
Seeing or hearing birds	1.53*** (1.37, 1.68)	1.07*** (0.90, 1.23)	1.72*** (1.53, 1.91)	1.22*** (1.01, 1.43)	1.72*** (1.42, 2.02)	1.25*** (0.92, 1.57)
Seeing or hearing birds * depression	− 0.26 (− 0.66, 0.13)	− 0.22 (− 0.61, 0.17)	− 0.24 (− 0.75, 0.26)	− 0.19 (− 0.69, 0.32)	− 0.18 (− 0.96, 0.61)	− 0.16 (− 0.95, 0.62)

Mean difference (MD) and 95% confidence intervals (CI) represent the mean difference in momentary mental wellbeing per category increase compared to the reference group.

Statistically significant associations (p < 0.05) are highlighted in bold.

Analyses were explored as crude associations and after adjusting for age, gender, ethnicity, education, occupation, whether a participant could see trees, plants, and see or hear water.

*p < 0.05.

**p < 0.01.

***p < 0.001.

### Is the beneficial impact of everyday encounters with birds on mental wellbeing time-lasting?

**Figure 2:**
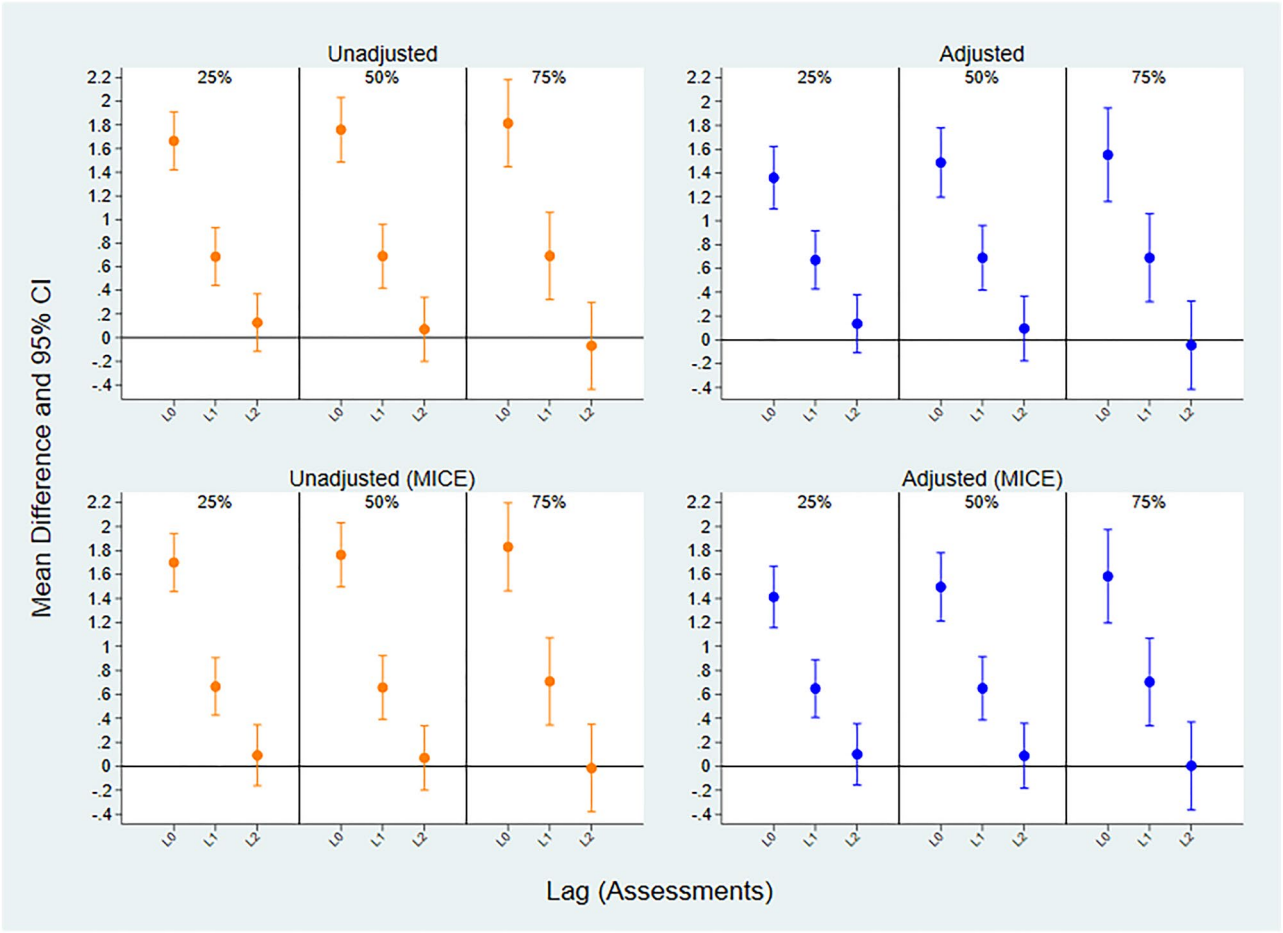
Time-lagged associations of momentary mental wellbeing scores in relation to seeing or hearing birds. Associations are shown unadjusted and adjusted for age, gender, education, occupational status, ethnicity, whether participants could see trees, plants, and see or hear water, and with the implementation of the MICE procedure. The vertical axis represents the mean difference (MD) and 95% confidence intervals (95% CI) of mental wellbeing score when seeing or hearing birds. The horizontal axis represents the assessments lagged from 0 to 2. L0 indicates the impact of seeing or hearing birds on mental wellbeing at the time of the assessment. L1 indicates the impact of seeing or hearing birds on mental wellbeing during the subsequent assessment. L2 indicates the impact of seeing or hearing birds on mental wellbeing in the second subsequent assessment.

Using time-lagged random intercept regression models, we found a positive association between seeing or hearing birds and momentary mental wellbeing during the subsequent assessment (_L1_MD: 0.69; 95% CI 0.42, 0.96). While the positive association between mental wellbeing and seeing or hearing birds remained during the subsequent assessment (L1), the effect was lower than at the time of exposure. The time-lasting effect was not evident during the second subsequent assessment (L2). These results were consistent across our sensitivity samples, when adjusting for potential confounders, when within-subject centering the predictor variable, when implementing the MICE procedures to address missing data, and when adjusting for the autoregressive effects of latent mean centered mental wellbeing at the prior timepoint (see Fig. 2, Supplementary Tables S2, S3, S4, and S9).

### Does the beneficial impact of everyday encounters with birds on mental wellbeing differ between people with and without a diagnosis of depression?

**Figure 3:**
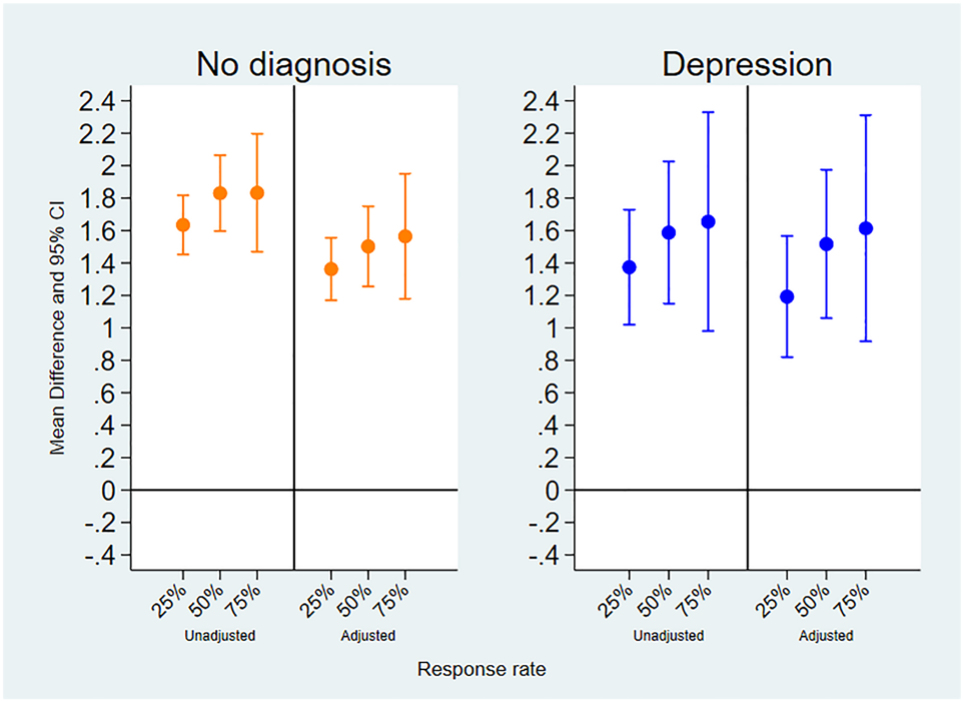
Associations of momentary mental wellbeing scores in relation to seeing or hearing birds in participants with no diagnosis of mental health condition and those with a diagnosis of depression. Associations are shown unadjusted and adjusted for age, gender, education, occupational status, ethnicity, whether participants could see trees, plants, and see or hear water. The vertical axis represents the mean difference (MD) and 95% confidence intervals (95% CI) of mental wellbeing score when seeing or hearing birds. The horizontal axis represents the assessments lagged from 0 to 2. L0 indicates the impact of seeing or hearing birds on mental wellbeing at the time of the assessment. L1 indicates the impact of seeing or hearing birds on mental wellbeing during the subsequent assessment. L2 indicates the impact of seeing or hearing birds on mental wellbeing in the second subsequent assessment.

In order to test whether the positive effect of everyday encounters with birds on mental wellbeing differs between people with a diagnosis of depression and people without a mental health condition, we included an interaction term into our multilevel regression models. When adjusting for age, gender, ethnicity, education, occupational status, whether a participant could see trees, plants, and see or hear water at the time of the assessments, having a diagnosis of depression did not interact with the association between seeing or hearing birds and mental wellbeing (*p* > 0.05) (See Table 2). This suggests that the positive effect of encounters with birds on mental wellbeing was remained consistent in people without a mental health condition and those who with a diagnosis of depression. Further subgroup analyses showed that the positive effect was evident in both people with depression and people without a mental health condition (See Fig. 3).

## Discussion

The main aim of the present study was to investigate the mental health benefits of encountering birds as part of everyday life. This was achieved using smartphone-based EMA, which allowed us to sample people’s experiences in real-time and real-world contexts.

Consistent with our first hypothesis, we found that participants’ mental wellbeing was significantly better when seeing or hearing birds compared to when not seeing or hearing birds. The effect was robust and observed at all three completion rate thresholds as well as after adjusting for potential sociodemographic confounders including age, gender, ethnicity, education and occupation. Interestingly, we found that the positive effect of seeing or hearing birds on mental wellbeing was more pronounced when individuals were outdoors (see Supplementary Table S8). People are most likely to see or hear birds in the context of green spaces, raising the possibility that the association between birdlife and mental wellbeing might in fact reflect an overall effect of nature on mental wellbeing. In order to minimise such possibility, we modelled seeing trees, plants, and seeing or hearing water as additional confounding variables. Critically, the results were still significant, providing support to a specific benefit of birdlife on mental wellbeing, above and beyond the well-established effect of green spaces.

Consistent with our second hypothesis, we found that the beneficial effect on mental wellbeing is still significant after the encounter with birds has taken place. This is consistent with our earlier research on the benefits of green^10^ and blue^18^ spaces which demonstrated time-lasting benefits for mental health. Perhaps unsurprisingly, the beneficial effect of seeing or hearing birds on mental wellbeing does wane over time. Specifically, it is reduced at the time of the first subsequent assessment following the encounter and is no longer significant at the time of the second subsequent assessment following the encounter. The use of an observational design meant that we were unable to establish causality in our findings. Nevertheless, we found that the association between seeing or hearing birds and mental wellbeing was still evident in the subsequent assessment, whereas mental wellbeing at a given timepoint did not increase the odds of seeing or hearing birds at subsequent timepoints (see Supplementary Tables S6, S7). This pattern of results could be an indication of a possible causal link effect of birdlife on mental wellbeing.

Consistent with our third hypothesis, the beneficial impact of everyday experience of birds on mental wellbeing was evident in both people with depression and people without a mental health condition. Once again, this finding was observed at all three completion rate thresholds as well as after adjusting for potential sociodemographic confounders including age, gender, ethnicity, education, occupation, seeing trees, plants and seeing or hearing water at the time of the assessment. Depression is the leading cause of disability and sick leave globally^19^, affecting an estimated 350 million people^20^. While antidepressant medication can lead to a significant reduction of symptoms^12^, there is an urgent need for non-pharmacological interventions to support mental health in people who have developed this illness. The current evidence for mental health benefits of green spaces in people with depression is mixed, with some studies reporting significant effects^21–23^ and other suggesting reduced benefits relative to healthy individuals^24,25^. Our investigation extends this existing literature by demonstrating that everyday experience of birdlife has beneficial effects not only in healthy individuals but also in people with a diagnosis of depression.

The potential policy implications of the present study are two-fold. First, the findings have implications for environmental and wildlife protection policy. The past few decades have seen a gradual but constant decline in biodiversity. A recent European report has revealed that there are 247 million fewer house sparrows and that one in six bird species have disappeared since the 1980s^26^. The reasons for this decline are complex. In rural areas, agricultural intensification and farming with chemicals are causing habitat loss and the disappearance of insects that feed birds; whereas in urban areas, bird population is falling due to a combination of trends including shortages of food, habitat loss, increase in diseases such as avian malaria and raising levels of air pollution. Our investigation provides support to the introduction of environmental and wildlife protection policies which encourage biodiverse habitats in urban, suburban and rural areas (e.g. permaculture farming, wilding initiatives, hedgerow and meadow enhancement, urban forestry). Second, the findings have implications for mental healthcare policy. In recent years social prescribing of nature-based activities, also known as “green prescribing”, has become increasingly popular to support individuals with mental illness including depression^27,28^. Our investigation supports the notion that visits to habitats with a high degree of birdlife, such as parks and canals, may be encouraged as part of green prescribing efforts.

### Strengths

Previous studies examined the potential mental health benefits of birdlife using surveys or questionnaires requiring participants to recollect past experiences, or an artificial experimental setting involving the presentation of bird-related images or sounds to people sitting in front of a computer screen. In the present study, the use of EMAs allowed us to capture dynamic changes in the participants’ whereabouts and mental wellbeing in real-time and in real-world contexts. In particular we used smartphone-based EMAs, which provide more accurate and complete measurements when compared to the traditional method of paper diaries and stand-alone electronic devices^29^.

While the use of an observational design means that we cannot be certain that the observed increases in mental wellbeing are due to seeing or hearing birds alone, our analyses were adjusted for known sociodemographic confounders (age, gender, ethnicity, education, and occupation) as well as exposure to trees, plants, and water at the time of the assessment. In addition, the fact that the observed increases in mental wellbeing are still evident after the encounter with birds has taken place provides indirect support a potential causal link.

### Limitations

The sample in this study was self-selected, recruited through a limited range of social media and websites. Furthermore, participants were aware of the fact that the study aimed to investigate the impact of the social and built environment on mental wellbeing, which may have made them more conscious about how they were feeling and bias their responses. While the 42-month recruitment timeframe allowed us to recruit a large sample, this occurred prior to and during the Covid-19 pandemic, which may have changed people’s stress levels and response to birdlife. Future studies should take potential effects of the pandemic into account. In addition, our sample still consisted primarily of white, university educated individuals based in the UK who were either in employment or education. Caution should hence be taken when applying the findings to the general population. Additionally, participants were asked to self-report whether they had ever been diagnosed with a mental health condition, and to indicate their diagnosis. Furthermore, while participants were allowed to select multiple diagnoses, the potential effects of comorbid diagnoses were not taken into account in the statistical analysis. Future studies would benefit from the use of validated clinical instruments to assess current symptoms and diagnosis, and the exploration of possible effects of comorbidity on findings. Finally, since we asked participants if they could see or hear birds, we were unable to dissociate between the potential mental health benefits of “seeing” and “hearing” birdlife. Nevertheless, in our encounters with birdlife, we do not perceive images and sounds of birds in a vacuum but as part of our multi-sensory experience. Therefore, it may be reductive to focus on visual and auditory aspects when assessing the mental health benefits of birdlife in real-time and real-world contexts. Consistent with this idea, a recent investigation suggests that the restorative potential of nature is greater when considering visual and auditory aspects together than when focussing on either modality separately^30^.

## Conclusions

To our knowledge, this is the first study examining the impact of everyday encounters with birds on mental wellbeing in real-time and real-life contexts. We report significant mental health benefits of birdlife, evident not only in healthy people but also in those with a diagnosis of depression. Further research on a more diverse sample is needed to allow the generalisation of these findings to the general population. Our study has potential for mental healthcare policy. Visits to habitats with a high degree of birdlife could become part of social prescribing schemes, playing a role in preventing mental health difficulties and complementing more traditional interventions. Paramount to all of this, will be the adoption of environmental and wildlife protection policies for the preservation and enhancement of a mosaic of habitats in rural and urban settings.

## Supplementary Information

Supplementary Information.

## Data Availability

The data generated and analysed for the current study are not publicly available due to further analyses being planned, but de-identified data may be made available from the corresponding author on reasonable request.
