# Supplementary material for: Smartphone-based ecological momentary assessment reveals mental health benefits of birdlife
Source: Sci Rep. 2022 Oct 27;12:17589. doi: 10.1038/s41598-022-20207-6 (PMC9614007; doi:10.1038/s41598-022-20207-6)
Supplement: Supplementary file 1 — Supplementary Information. [file 41598_2022_20207_MOESM1_ESM.docx]

**Supplementary Tables:**

**Supplementary Table 1.** Mean difference (MD) between seeing or hearing birds and mental wellbeing at the 25%, 50% and 75% response rates using the Multiple Imputation with Chains Equations (MICE) procedure.

|  | **25% response rate (*n* = 1292)** | | **50% response rate (*n* = 593)** | | **75% response rate (*n* = 196)** | |
| --- | --- | --- | --- | --- | --- | --- |
|  | Unadjusted | Adjusted | Unadjusted | Adjusted | Unadjusted | Adjusted |
|  | **MD**  **(95% CI)** | **MD**  **(95% CI)** | **MD**  **(95% CI)** | **MD**  **(95% CI)** | **MD**  **(95% CI)** | **MD**  **(95% CI)** |
| Seeing or hearing birds | **1.57*****  **(1.39, 1.75)** | **1.09*****  **(0.87, 1.30)** | **1.75*****  **(1.55, 1.94)** | **1.22*****  **(1.01, 1.43)** | **1.77*****  **(1.42, 2.12)** | **1.27*****  **(0.91, 1.63)** |
| Seeing or hearing birds * Depression | -0.30  (-0.77, 0.18) | -0.25  (-0.73, 0.22) | -0.36  (-0.91, 0.19) | -0.28  (-0.83, 0.26) | -0.21  (-1.02, 0.61) | -0.18  (-0.99, 0.63) |
| *Note: Mean difference (MD) and 95% confidence intervals (CI) represent the mean difference in momentary mental wellbeing per category increase compared to the reference group. Statistically significant associations (p* < 0.05*) are highlighted in bold.*  *All models employed the Multiple Imputation with Chained Equations (MICE) procedure. Analyses were explored as crude associations and after adjusting for age, gender, ethnicity, education, occupation, whether a participant could see trees, plants, and see or hear water.*  ** p* < 0.05  *** p* < 0.01  **** p* < 0.001 | | | | | | |

**Supplementary Table 2.** Time-lagged associations between seeing or hearing birds and mental wellbeing at the 25%, 50% and 75% response rates.

|  | **25% response rate (*n* = 1292)** | | | **50% response rate (*n* = 593)** | | **75% response rate (*n* = 196)** | |
| --- | --- | --- | --- | --- | --- | --- | --- |
|  | Unadjusted | | Adjusted | Unadjusted | Adjusted | Unadjusted | Adjusted |
|  | **MD**  **(95% CI)** | | **MD**  **(95% CI)** | **MD**  **(95% CI)** | **MD**  **(95% CI)** | **MD**  **(95% CI)** | **MD**  **(95% CI)** |
| Seeing or hearing birds | |  |  |  |  |  |  |
| L0 | **1.67*****  **(1.42, 1.91)** | | **1.15*****  **(0.89, 1.42)** | **1.76*****  **(1.49, 2.03)** | **1.24*****  **(0.93, 1.53)** | **1.81*****  **(1.45, 2.18)** | **1.30*****  **(0.90, 1.70)** |
| L1 | **0.69*****  **(0.44, 0.93)** | | **0.63****  **(0.38, 0.87)** | **0.69*****  **(0.42, 0.96)** | **0.65*****  **(0.38, 0.92)** | **0.69*****  **(0.32, 1.06)** | **0.65****  **(0.28, 1.02)** |
| L2 | 0.13  (-0.12, 0.37) | | 0.12  (-0.12, 0.37) | 0.07  (-0.20, 0.34) | 0.09  (-0.18, 0.36) | -0.07  (-0.44, 0.30) | -0.05  (-0.42, 0.12) |
| *Note: Mean difference (MD) and 95% confidence intervals (CI) represent the mean difference in momentary mental wellbeing per category increase compared to the reference group. Statistically significant associations (p* < 0.05*) are highlighted in bold.*  *L0 indicates the impact of seeing or hearing birds on mental wellbeing at the time of the assessment. L1 indicates the impact of seeing or hearing birds on mental wellbeing during the subsequent assessment. L2 indicates the impact of seeing or hearing birds on mental wellbeing in the second subsequent assessment.*  *Analyses were explored as crude associations and after adjusting for age, gender, ethnicity, education, occupation, whether a participant could see trees, plants, and see or hear water.*  ** p* < 0.05  *** p* < 0.01  **** p* < 0.001 | | | | | | | |

**Supplementary Table 3.** Time-lagged associations between seeing or hearing birds and mental wellbeing at the 25%, 50% and 75% response rates using the Multiple Imputation with Chains Equations (MICE) procedure.

|  | **25% response rate (*n* = 1292)** | | | **50% response rate (*n* = 593)** | | **75% response rate (*n* = 196)** | |
| --- | --- | --- | --- | --- | --- | --- | --- |
|  | Unadjusted | | Adjusted | Unadjusted | Adjusted | Unadjusted | Adjusted |
|  | **MD**  **(95% CI)** | | **MD**  **(95% CI)** | **MD**  **(95% CI)** | **MD**  **(95% CI)** | **MD**  **(95% CI)** | **MD**  **(95% CI)** |
| Seeing or hearing birds | |  |  |  |  |  |  |
| L0 | **1.71*****  **(1.46, 1.95)** | | **1.21*****  **(0.95, 1.47)** | **1.81*****  **(1.54, 2.08)** | **1.31*****  **(1.01, 1.60)** | **1.83*****  **(1.46, 2.19)** | **1.33*****  **(0.93, 1.72)** |
| L1 | **0.67*****  **(0.43, 0.91)** | | **0.60*****  **(0.36, 0.84)** | **0.66*****  **(0.39, 0.93)** | **0.61*****  **(0.34, 0.88)** | **0.70*****  **(0.34, 1.07)** | **0.66*****  **(0.30, 1.02)** |
| L2 | 0.11  (-0.14, 0.35) | | 0.10  (-0.14, 0.35) | 0.05  (-0.22, 0.32) | 0.07  (-0.20, 0.34) | -0.04  (-0.41, 0.33) | -0.03  (-0.41, 0.34) |
| *Note: Mean difference (MD) and 95% confidence intervals (CI) represent the mean difference in momentary mental wellbeing per category increase compared to the reference group. Statistically significant associations (p* < 0.05*) are highlighted in bold.*  *L0 indicates the impact of seeing or hearing birds on mental wellbeing at the time of the assessment. L1 indicates the impact of seeing or hearing birds on mental wellbeing during the subsequent assessment. L2 indicates the impact of seeing or hearing birds on mental wellbeing in the second subsequent assessment.*  *All models employed the Multiple Imputation with Chained Equations (MICE) procedure.*  *Analyses were explored as crude associations and after adjusting for age, gender, ethnicity, education, occupation, whether a participant could see trees, plants, and see or hear water.*  ** p* < 0.05  *** p* < 0.01  **** p* < 0.001 | | | | | | | |

**Supplementary Table 4.** Mean difference (MD), interactions, and time-lagged associations between seeing or hearing birds and mental wellbeing at the 25%, 50% and 75% response after within-subject centring the exposure variable.

|  | **25% response rate (*n* = 1292)** | | | **50% response rate (*n* = 593)** | | **75% response rate (*n* = 196)** | |
| --- | --- | --- | --- | --- | --- | --- | --- |
|  | Unadjusted | | Adjusted | Unadjusted | Adjusted | Unadjusted | Adjusted |
|  | **MD**  **(95% CI)** | | **MD**  **(95% CI)** | **MD**  **(95% CI)** | **MD**  **(95% CI)** | **MD**  **(95% CI)** | **MD**  **(95% CI)** |
| Seeing or hearing birds | **1.50*****  **(1.34, 1.65)** | | **1.04*****  **(0.87, 1.20)** | **1.70*****  **(1.51, 1.90)** | **1.21*****  **(1.00, 1.42)** | **1.69*****  **(1.39, 2.00)** | **1.23*****  **(0.91, 1.55)** |
| Seeing or hearing birds * Depression | -0.24  (-0.63, 0.15) | | -0.20  (-0.60, 0.19) | -0.19  (-0.70, 0.31) | -0.14  (-0.64, 0.37) | -0.13  (-0.91, 0.66) | -0.11  (-0.90, 0.67) |
| Seeing or hearing birds | |  |  |  |  |  |  |
| L0 | **1.61*****  **(1.36, 1.86)** | | **1.11*****  **(0.85, 1.38)** | **1.73*****  **(1.46, 2.01)** | **1.22*****  **(0.92, 1.51)** | **1.80*****  **(1.41, 2.15)** | **1.27*****  **(0.87, 1.67)** |
| L1 | **0.63*****  **(0.38, 0.88)** | | **0.60*****  **(0.36, 0.85)** | **0.66*****  **(0.39, 0.94)** | **0.64*****  **(0.36, 0.91)** | **0.66*****  **(0.29, 1.03)** | **0.63****  **(0.26, 1.00)** |
| L2 | 0.07  (-0.17, 0.32) | | 0.10  (-0.15, 0.35) | 0.04  (-0.23, 0.32) | 0.08  (-0.19, 0.36) | -0.10  (-0.48, 0.27) | -0.07  (-0.44, 0.30) |
| *Note: Mean difference (MD) and 95% confidence intervals (CI) represent the mean difference in momentary mental wellbeing per category increase compared to the reference group. Statistically significant associations (p* < 0.05*) are highlighted in bold.*  *All models utilised within-subject centred exposure variables. Analyses were explored as crude associations and after adjusting for age, gender, ethnicity, education, occupation, whether a participant could see trees, plants, and see or hear water.*  ** p* < 0.05  *** p* < 0.01  **** p* < 0.001 | | | | | | | |

**Supplementary Table 5.** Mean difference (MD) between seeing or hearing birds and mental wellbeing at the 25%, 50% and 75% response rates utilising random slopes of the focal exposure in the models.

|  | **25% response rate (*n* = 1292)** | | **50% response rate (*n* = 593)** | | **75% response rate (*n* = 196)** | |
| --- | --- | --- | --- | --- | --- | --- |
|  | Unadjusted | Adjusted | Unadjusted | Adjusted | Unadjusted | Adjusted |
|  | **MD**  **(95% CI)** | **MD**  **(95% CI)** | **MD**  **(95% CI)** | **MD**  **(95% CI)** | **MD**  **(95% CI)** | **MD**  **(95% CI)** |
| Seeing or hearing birds | **1.50*****  **(1.31, 1.70)** | **1.06*****  **(0.86, 1.27)** | **1.75*****  **(1.48, 2.02)** | **1.27*****  **(0.99, 1.54)** | **1.77*****  **(1.32, 2.22)** | **1.29*****  **(0.83, 1.74)** |
| Seeing or hearing birds * Depression | -0.20  (-0.71, 0.30) | -0.18  (-0.67, 0.32) | -0.16  (-0.87, 0.54) | -0.13  (-0.82, 0.55) | -0.18  (-1.36, 1.00) | -0.17  (-1.33, 0.99) |
| *Note: Mean difference (MD) and 95% confidence intervals (CI) represent the mean difference in momentary mental wellbeing per category increase compared to the reference group. Statistically significant associations (p* < 0.05*) are highlighted in bold.*  *All models included random slopes of the focal exposure. Analyses were explored as crude associations and after adjusting for age, gender, ethnicity, education, occupation, whether a participant could see trees, plants, and see or hear water.*  ** p* < 0.05  *** p* < 0.01  **** p* < 0.001 | | | | | | |

**Supplementary Table 6.** Time-lagged associations exploring whether mental wellbeing is associated with seeing or hearing birds during subsequent timepoints at the 25%, 50% and 75% response rates.

|  | **25% response rate (*n* = 1292)** | | | **50% response rate (*n* = 593)** | | **75% response rate (*n* = 196)** | |
| --- | --- | --- | --- | --- | --- | --- | --- |
|  | Unadjusted | | Adjusted | Unadjusted | Adjusted | Unadjusted | Adjusted |
|  | **OR**  **(95% CI)** | | **OR**  **(95% CI)** | **OR**  **(95% CI)** | **OR**  **(95% CI)** | **OR**  **(95% CI)** | **OR**  **(95% CI)** |
| Mental Wellbeing | |  |  |  |  |  |  |
| L0 | **1.07*****  **(1.06, 1.08)** | | **1.04*****  **(1.03, 1.06)** | **1.08*****  **(1.06, 1.09)** | **1.05*****  **(1.03, 1.06)** | **1.07*****  **(1.06, 1.09)** | **1.05*****  **(1.03, 1.07)** |
| L1 | **0.99***  **(0.98, 1.00)** | | 0.99  (0.98, 1.00) | **0.99***  **(0.98, 1.00)** | 0.99  (0.98, 1.00) | 0.99  (0.98, 1.01) | 1.00  (0.98, 1.02) |
| L2 | 1.00  (0.99, 1.02) | | 1.00  (0.99, 1.01) | 1.01  (0.99, 1.02) | 1.01  (0.99, 1.02) | 1.01  (0.99, 1.02) | 1.00  (0.99, 1.02) |
| *Note: Odds ratio (OR) and 95% confidence intervals (CI) represent the odds ratio of seeing or hearing birds per unit increase of mental wellbeing. Statistically significant associations (p* < 0.05*) are highlighted in bold.*  *L0 indicates the impact of mental wellbeing on seeing or hearing birds at the time of the assessment. L1 indicates the impact of mental wellbeing on seeing or hearing birds during the subsequent assessment. L2 indicates the impact of mental wellbeing on seeing or hearing in the second subsequent assessment.*  *Analyses were explored as crude associations and after adjusting for age, gender, ethnicity, education, occupation, whether a participant could see trees, plants, and see or hear water.*  ** p* < 0.05  *** p* < 0.01  **** p* < 0.001 | | | | | | | |

**Supplementary Table 7.** Time-lagged associations exploring whether mental wellbeing is associated with seeing or hearing birds during subsequent timepoints at the 25%, 50% and 75% response rates using the Multiple Imputation with Chains Equations (MICE) procedure.

|  | **25% response rate (*n* = 1292)** | | | **50% response rate (*n* = 593)** | | **75% response rate (*n* = 196)** | |
| --- | --- | --- | --- | --- | --- | --- | --- |
|  | Unadjusted | | Adjusted | Unadjusted | Adjusted | Unadjusted | Adjusted |
|  | **OR**  **(95% CI)** | | **OR**  **(95% CI)** | **OR**  **(95% CI)** | **OR**  **(95% CI)** | **OR**  **(95% CI)** | **OR**  **(95% CI)** |
| Mental Wellbeing | |  |  |  |  |  |  |
| L0 | **1.07*****  **(1.06, 1.08)** | | **1.04*****  **(1.03, 1.06)** | **1.08*****  **(1.06, 1.09)** | **1.05*****  **(1.04, 1.06)** | **1.07*****  **(1.06, 1.09)** | **1.05*****  **(1.03, 1.07)** |
| L1 | **0.99***  **(0.98, 1.00)** | | 0.99  (0.98, 1.00) | 0.99  (0.98, 1.00) | 0.99  (0.98, 1.00) | 0.99  (0.98, 1.01) | 1.00  (0.98, 1.02) |
| L2 | 1.00  (1.00, 1.01) | | 1.00  (0.99, 1.01) | 1.01  (0.99, 1.02) | 1.01  (0.99, 1.02) | 1.01  (0.99, 1.02) | 1.00  (0.99, 1.02) |
| *Note: Odds ratio (OR) and 95% confidence intervals (CI) represent the odds ratio of seeing or hearing birds per unit increase of mental wellbeing. Statistically significant associations (p* < 0.05*) are highlighted in bold.*  *L0 indicates the impact of mental wellbeing on seeing or hearing birds at the time of the assessment. L1 indicates the impact of mental wellbeing on seeing or hearing birds during the subsequent assessment. L2 indicates the impact of mental wellbeing on seeing or hearing in the second subsequent assessment.*  *All models employed the Multiple Imputation with Chained Equations (MICE) procedure.*  *Analyses were explored as crude associations and after adjusting for age, gender, ethnicity, education, occupation, whether a participant could see trees, plants, and see or hear water.*  ** p* < 0.05  *** p* < 0.01  **** p* < 0.001 | | | | | | | |

**Supplementary Table 8.** Interaction effect of being outdoors on the association between seeing or hearing birds and mental wellbeing at the 25%, 50% and 75% response rates.

|  | **25% response rate (*n* = 1292)** | | **50% response rate (*n* = 593)** | | **75% response rate (*n* = 196)** | |
| --- | --- | --- | --- | --- | --- | --- |
|  | Unadjusted | Adjusted | Unadjusted | Adjusted | Unadjusted | Adjusted |
|  | **MD**  **(95% CI)** | **MD**  **(95% CI)** | **MD**  **(95% CI)** | **MD**  **(95% CI)** | **MD**  **(95% CI)** | **MD**  **(95% CI)** |
| Seeing or hearing birds * Outdoors | **1.28*****  **(0.94, 1.61)** | **1.23*****  **(0.89, 1.56)** | **1.29*****  **(0.86, 1.71)** | **1.24*****  **(0.81, 1.67)** | **1.84*****  **(1.18, 2.51)** | **1.78*****  **(1.11, 2.45)** |
| *Note: Mean difference (MD) and 95% confidence intervals (CI) represent the mean difference in momentary mental wellbeing per category increase compared to the reference group. Statistically significant associations (p* < 0.05*) are highlighted in bold.*  *Analyses were explored as crude associations and after adjusting for age, gender, ethnicity, education, occupation, whether a participant could see trees, plants, and see or hear water.*  ** p* < 0.05  *** p* < 0.01  **** p* < 0.001 | | | | | | |

**Supplementary Table 9.** Time-lagged associations between seeing or hearing birds and mental wellbeing at the 25%, 50% and 75% response rates adjusted for the autoregressive effects of mental wellbeing.

|  | **25% response rate (*n* = 1292)** | | | **50% response rate (*n* = 593)** | | **75% response rate (*n* = 196)** | |
| --- | --- | --- | --- | --- | --- | --- | --- |
|  | Unadjusted | | Adjusted | Unadjusted | Adjusted | Unadjusted | Adjusted |
|  | **MD**  **(95% CI)** | | **MD**  **(95% CI)** | **MD**  **(95% CI)** | **MD**  **(95% CI)** | **MD**  **(95% CI)** | **MD**  **(95% CI)** |
| Seeing or hearing birds | |  |  |  |  |  |  |
| L0 | **1.66*****  **(1.42, 1.90)** | | **1.15*****  **(0.89, 1.41)** | **1.73*****  **(1.47, 2.00)** | **1.21*****  **(0.92, 1.50)** | **1.76*****  **(1.40, 2.12)** | **1.24*****  **(0.85, 1.63)** |
| L1 | **0.40****  **(0.15, 0.64)** | | **0.34****  **(0.09, 0.58)** | **0.33***  **(0.07, 0.60)** | **0.29***  **(0.02, 0.56)** | 0.30  (-0.06, 0.66) | 0.25  (-0.11, 0.62) |
| L2 | 0.07  (-0.17, 0.32) | | 0.07  (-0.17, 0.31) | -0.01  (-0.28, 0.25) | 0.01  (-0.26, 0.27) | -0.19  (-0.55, 0.18) | -0.17  (-0.53, 0.19) |
| *Note: Mean difference (MD) and 95% confidence intervals (CI) represent the mean difference in momentary mental wellbeing per category increase compared to the reference group. Statistically significant associations (p* < 0.05*) are highlighted in bold.*  *L0 indicates the impact of seeing or hearing birds on mental wellbeing at the time of the assessment. L1 indicates the impact of seeing or hearing birds on mental wellbeing during the subsequent assessment. L2 indicates the impact of seeing or hearing birds on mental wellbeing in the second subsequent assessment.*  *Analyses were explored as crude associations adjusted for autoregressive effects of latent centered mental wellbeing at the prior timepoint and after adjusting for age, gender, ethnicity, education, occupation, whether a participant could see trees, plants, and see or hear water.*  ** p* < 0.05  *** p* < 0.01  **** p* < 0.001 | | | | | | | |
